# Effectiveness of mechanical vibration-assisted sputum clearance in pediatric pneumonia: a randomized controlled trial with development of an intelligent parameter optimization model

**DOI:** 10.3389/fped.2025.1539218

**Published:** 2025-06-26

**Authors:** Sujing Xue, Kewu Lin, Ledan Lin, Huijie Zhou, Lishuang Dong, Huafen Chen, Tengfei Zhang

**Affiliations:** ^1^ Department of Pediatric Internal Medicine, Wenzhou People’s Hospital, Wenzhou Maternal and Child Health Care Hospital, Wenzhou, Zhejiang, China; ^2^ Department of Nursing, Wenzhou People’s Hospital, Wenzhou Maternal and Child Health Care Hospital, Wenzhou, Zhejiang, China

**Keywords:** pediatric pneumonia, mechanical vibration, sputum clearance, optimizing parameters, nomogram

## Abstract

**Background:**

Sputum retention in pediatric pneumonia hinders recovery. This study evaluated the therapeutic benefits of incorporating mechanical vibration-assisted sputum clearance (MVSC) as an adjunct to standard pharmacological treatment and developed a nomogram to optimize treatment parameters.

**Methods:**

In this randomized controlled trial, 160 children with pneumonia were randomly assigned (1:1) to a control group receiving pharmacological treatment or a treatment group receiving additional mechanical vibration therapy. The proportion of patients showing clinical improvement (defined as “markedly improved” or “improved”) was assessed, along with symptom resolution times, lung function, blood gas parameters, and inflammatory markers, which were measured at baseline and treatment completion. LASSO regression identified predictors of clinical improvement.

**Results:**

A significantly greater proportion of patients exhibited clinical improvement in the treatment group (93.75%) compared to the control group (75%, *P* < 0.05). The treatment group also showed significantly shorter times for symptom resolution (pulmonary rales, fever, and cough) and hospital stay (*P* < 0.05). Improvements in lung function (FEV1, FEV1/FVC), blood gas parameters (PaO₂, PaCO₂, pH), and greater reductions in inflammatory markers (CRP, PCT), were observed in the treatment group (*P* < 0.05). A nomogram was developed using age, chest circumference, moist rales, disease severity, vibration frequency, and pressure to determine optimal mechanical vibration settings for individualized treatment. The nomogram showed high predictive accuracy (AUC = 0.943) in estimating clinical improvement, aiding in the optimization of MVSC in pediatric pneumonia.

**Conclusions:**

MVSC enhances the treatment of pediatric pneumonia, with the nomogram optimizing the parameters of the sputum clearance device.

## Introduction

Pediatric pneumonia is one of the most common respiratory diseases in children, characterized by a high incidence rate and significant health risks. Its clinical features include fever, cough, shortness of breath, and increased sputum production (1). Young children are particularly prone to sputum retention due to their immature respiratory systems, narrow airways, and insufficient self-clearing ability. This sputum retention can worsen the disease and even lead to severe complications (2). Traditional sputum clearance methods, such as postural drainage and chest percussion, are widely used but have limitations, including dependence on manual techniques and inconsistent therapeutic effects (3). Additionally, these methods require considerable technical expertise and patient cooperation, making it difficult to achieve precise and efficient treatment. In response to these challenges, there is growing interest in exploring individualized sputum clearance strategies, including respiratory physiotherapy. While not yet a routine practice for pediatric pneumonia in many healthcare systems (4), it has potential benefits as an adjunctive therapy in improving airway clearance (5).

In recent years, MVSC has gained attention in clinical applications. This provides an alternative to manually delivered chest percussions, allowing patients to perform them by wearing vest-like devices while positioned in the most comfortable postures, such as semi-recumbent or seated (6). The mechanism of action includes: (1) altering the rheological properties of secretions (7), (2) generating high-frequency oscillatory airflow that promotes the detachment of mucus from the airway walls (8), and (3) accelerating the movement of the ciliary system to expedite the transfer and expulsion of secretions to the larger airways (9). Mechanical vibration delivers specific frequency and amplitude stimuli to the thorax, effectively loosening and mobilizing sputum. It offers advantages such as being non-invasive, safe, and highly reproducible (10). However, challenges remain in the clinical application. First, the absence of standardized clinical guidelines often leads to the use of uniform and simplistic settings for vibration frequency and pressure (11). These fixed parameters fail to consider the wide variation in developmental stages among children, such as differences in chest size and physiological conditions (12), resulting in no improvement in sputum clearance for some patients (13). Second, the lack of intelligent models for parameter selection hinders the ability to tailor treatments to individual patient needs, reducing the potential effectiveness and precision of the therapy.

Therefore, this study aimed to evaluate the clinical efficacy of MVSC as an adjunct to standard pharmacological treatment in children with pneumonia. In addition, we developed a predictive nomogram model to estimate the probability of clinical improvement in children receiving MVSC therapy, based on both patient-specific characteristics and treatment parameters. This predictive tool can help clinicians optimize machine settings tailored to each child's physiological profile, thereby enhancing the precision and effectiveness of MVSC.

## Methods

### Trial design

This study was a single-center, prospective, randomized controlled trial (RCT). The study employed a parallel-group design, with participants randomly assigned to either the treatment group or the control group in a 1:1 ratio. The study followed the Consolidated Standards of Reporting Trials (CONSORT) guidelines.

This study not only evaluated the efficacy of MVSC in treating pediatric pneumonia but also introduced an intelligent parameter optimization approach. Using patient characteristics such as age, chest circumference, and disease severity, a predictive nomogram was constructed to identify optimal vibration settings for improved clinical outcomes. This model provided a personalized, evidence-based framework for parameter selection, aiming to enhance therapeutic efficacy and efficiency in the management of pediatric pneumonia.

### Ethical statements

The study followed the Declaration of Helsinki and was approved by the Ethics Committee of Wenzhou People's Hospital (approval number: KY-202408-024). The trial was registered on the Chinese Clinical Trial Registry (https://www.chictr.org.cn/) with the registration number: ChiCTR2400090484. Before the start of the study, the parents or guardians of the patients were fully informed about the purpose, methods, and potential risks of the study, and written informed consent was obtained after their agreement.

### Study population

The study included children diagnosed with pneumonia at Wenzhou People's Hospital from October 2024 to December 2024.

### Patients

Inclusion criteria:

(1) Age between 1 and 14 years.
(2) Good compliance and ability to cooperate with treatment.
(3) Diagnosis of pneumonia was based on clinical symptoms, imaging findings (e.g., chest x-ray or CT), and laboratory-confirmed pathogen detection (e.g., sputum culture, blood tests, or nucleic acid tests).
(4) Parent's/guardian's consent to participate in the study.

Exclusion criteria:

(1) Patients had significant comorbid conditions.
(2) Contraindications: ① Presence of vascular malformations; ② Pneumothorax or chest wall diseases; ③ Pulmonary thrombosis; ④ Pulmonary hemorrhage or hemoptysis; ⑤ Children who cannot tolerate vibration; ⑥ Skin infections at the site of instrument contact; ⑦ Abnormal coagulation mechanisms.
(3) Patients already participating in other clinical trials.

### Randomization and blinding

Eligible participants were randomly assigned to the treatment group or the control group in a 1:1 ratio. Randomization was performed by an independent statistician who was not involved in the study using SPSS 26.0 software. The randomization sequence was sealed in opaque, numbered envelopes, which were distributed to the families of the participants. Allocation was determined based on the random number in the envelope, ensuring unbiased group assignment.

To minimize potential bias, a single-blind design was employed. While blinding the caregivers and patients was not feasible due to the nature of the intervention, outcome assessors and data analysts remained blinded to group assignments throughout the study. Additionally, the clinical staff responsible for administering the intervention did not participate in data analysis.

### Intervention measures

**Figure 1:**
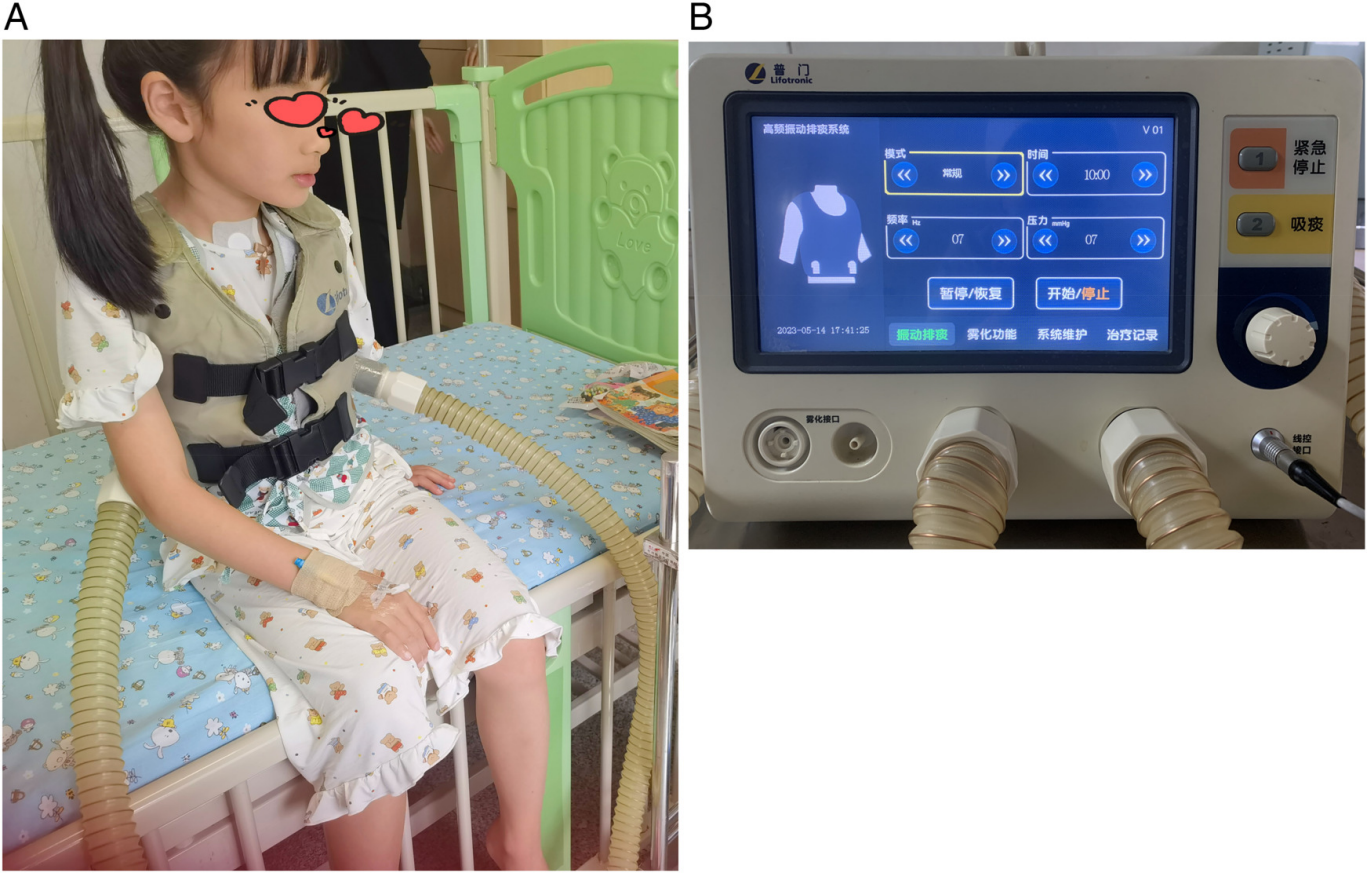
Mechanical sputum discharge instrument. **(A)** Physical picture of a wearable expectorator vest. **(B)** Operation interface for parameter adjustment of sputum discharge instrument.

In the control group, patients received standard pharmacological treatment, including antibiotics and symptomatic relief medications as per routine clinical guidelines. In the treatment group, patients received both pharmacological treatment and MVSC therapy (Figure 1). During parameter adjustment, patient comfort and tolerance were assessed in real-time by clinicians through clinical observation and communication with the children and their caregivers. Parameters were adjusted to the highest tolerable levels that did not cause discomfort. However, no standardized scale or formal quantitative assessment tool was employed to evaluate vibration tolerance in this study.

Adjustments were based on age, chest circumference, clinical signs (presence of moist rales), and disease severity (mild or severe). The device was initially set to a standard mode, but adjustments were made based on individual patient characteristics. Specific adjustment standards are shown in Table 1. The therapy was administered once daily, with each session lasting 10 min. The treatment course of both groups was 5 days, and the therapeutic effect was observed. Throughout the study, treatment compliance was closely monitored, and no deviations from the intervention protocol were observed.

**Table 1:** The adjustment standards for the mechanical vibration therapy.

Age	Chest circumference (cm)	Frequency (Hz)	Pressure (mmHg)	Adjustment based on clinical signs
1–3	50–60	4–8	3–8	Mild disease: lower-mid range settings; Severe disease: highest tolerable level.
4–8	60–70	5–10	6–10	Mild disease: lower-mid range settings; Severe disease: highest tolerable level.
9–14	70–80	6–12	7–12	Mild disease: lower-mid range settings; Severe disease: highest tolerable level.

### Sample size calculation

Currently, no studies have specifically investigated the application of this mechanical vibration-assisted sputum drainage device in pediatric pneumonia. Therefore, the sample size estimation was guided by clinical studies evaluating similar physical vibration therapies for pediatric pneumonia (14), as well as the expected number of recruited patients during the study period.

Using a two-sided test with an expected clinical efficacy rate of 70% in the control group and 90% in the treatment group, a significance level of α = 0.05, and a power of 1−β = 0.9 (90%), the required sample size was calculated using PASS 15.0 software. The calculation indicated that 64 participants per group. Considering an estimated dropout rate of 20%, the sample size was increased to 77 participants per group. To further ensure the stability and reliability of the study results, the final sample size was adjusted to 80 participants per group, resulting in a total of 160 patients. This adjustment accounted for potential variability and ensured the robustness of the statistical analysis.

### Data collection

The following indicators were collected at two time points: on Day 0 (upon hospital admission, before treatment initiation) and on Day 5 (after completion of the treatment period).

(1) To compare the baseline characteristics of the two groups, including gender, age, days of infection (defined as the number of days from the first appearance of symptoms, such as fever or cough, to hospital admission, as recorded by the attending physician during patient enrollment), respiratory rate, heart rate, chest circumference, moist rales, and disease severity at baseline (before treatment initiation, Day 0).
(2) To compare the levels of blood gas analysis indicators between the two groups at baseline (Day 0) and after treatment completion (Day 5). Blood gas analysis was performed using a blood gas biochemical analyzer, measuring pH, arterial oxygen partial pressure (PaO₂), and arterial carbon dioxide partial pressure (PaCO₂).
(3) To compare the levels of inflammatory factors between the two groups at baseline (Day 0) and after treatment completion (Day 5). The levels of C-reactive protein (CRP) and procalcitonin (PCT) in venous blood were measured using an enzyme-linked immunosorbent assay (ELISA).
(4) To compare the lung function indicators between the two groups at baseline (Day 0) and after treatment completion (Day 5), including forced expiratory volume in 1 s (FEV1) and the ratio of FEV1 to forced vital capacity (FEV1/FVC). These tests were performed only in children aged ≥5 years, as younger children were generally unable to complete reliable spirometry.
(5) To compare the improvement in clinical symptoms between the two groups. Symptom resolution times were recorded daily by attending physicians and nursing staff from Day 1 to Day 5.

Disappearance time of cough: Defined as the first recorded day on which the patient no longer exhibited cough symptoms, confirmed through physician assessment and caregiver reports.

Disappearance time of pulmonary rales: Assessed by auscultation twice daily (morning and evening). A disappearance was confirmed when no abnormal lung sounds were detected for two consecutive assessments by two independent pediatricians specializing in respiratory diseases.

Fever resolution time: Monitored every 6 h, defined as maintaining a normal body temperature (<37.5°C) for at least 24 h without the use of antipyretic medications.

Length of stay: Defined as the number of days from admission to discharge, recorded upon patient discharge based on meeting discharge criteria.

### Clinical efficacy evaluation

In this study, clinical efficacy referred to the degree of symptom improvement, resolution of pulmonary signs, and radiographic changes following treatment. The evaluation criteria were applied uniformly to both the treatment and control groups. The definitions were consistent with previously established evaluation standards in pediatric pneumonia studies (15). The criteria were as follows:

Markedly effective (markedly improved): Symptoms resolved, pulmonary rales disappeared, and chest x-ray showed absorption of pulmonary lesions.

Effective (improved): Symptoms alleviated, pulmonary rales reduced, but chest x-ray showed incomplete absorption of pulmonary lesions.

Ineffective: No improvement or worsening of symptoms and clinical signs.

Effective rate (%) = [(Number of patients with “Markedly effective” + “Effective”)/Total number of patients] × 100%.

Chest x-ray findings were assessed by a radiologist. The assessment focused on lesion absorption and improvement in lung opacity.

### Data preprocessing

Before analysis, data cleaning procedures were conducted. Variables with more than 30% missing data were excluded from the analysis. For variables with less than 30% missing data, multiple imputation techniques were applied to preserve the integrity of the dataset. In addition, to facilitate analysis, several variables were categorized as follows:

1. Moist rales
  (1) Few: Sparse crackles heard in a limited lung field, labeled as 1.
  (2) Moderate: Diffuse crackles heard in multiple lung fields but not throughout the entire lung, labeled as 2.
  (3) Many: Extensive crackles present in nearly all lung fields, labeled as 3._
2. Disease severity
  Disease severity was classified based on existing standards, such as the British Thoracic Society (BTS) guidelines (16). According to these guidelines, pediatric pneumonia is categorized as either mild or severe:
  (1) Mild pneumonia, labeled as 1.
  (2) Severe pneumonia, labeled as 2.
3 Gender
  Female labeled as 0. Male labeled as 1.
4 Age classification
  1–3 years labeled as 1. 4–8 years labeled as 2. 9–14 years labeled as 3.

### Variable selection and model construction

In the treatment group receiving MVSC therapy, the least absolute shrinkage and selection operator (LASSO) regression was employed to identify the most predictive variables for treatment outcomes from 10 candidate features: gender, days of infection, respiratory rate, heart rate, age classification, chest circumference, moist rales, disease severity, vibration frequency, and vibration pressure. LASSO is a logistic regression model that applies a penalty to variable coefficients, effectively reducing the coefficients of less relevant variables to zero and retaining only the most important predictors in the model. Cross-validation was used to determine the optimal regularization parameter for the LASSO model (17).

Subsequently, a logistic regression model was constructed using the variables selected through LASSO regression. The dependent variable was defined as whether the comprehensive clinical outcome of the treatment group improved. The comprehensive outcome was defined as follows:

(1) Improved: Included patients classified as “Markedly Effective” and “Effective”.
(2) Not Improved: Corresponds to “Ineffective” patients, meaning no improvement or worsening of symptoms and clinical signs.

A nomogram was developed to visualize the results, and the predictive performance of the model was evaluated in terms of discrimination, calibration, and clinical utility. Discrimination was assessed using the area under the receiver operating characteristic curve (AUC-ROC), while calibration was evaluated using a calibration plot. Decision curve analysis (DCA) was used to assess the clinical utility of the nomogram by calculating the net benefit at different threshold probabilities. A clinical impact curve (CIC) was used to evaluate the effectiveness of clinical predictions.

Application of the nomogram: By inputting known patient characteristics (e.g., chest circumference, age, disease severity, and extent of moist rales), the nomogram can help clinicians identify the optimal range of device frequency and pressure that are associated with a higher probability of clinical improvement. This allows for individualized adjustment of mechanical vibration parameters based on predicted treatment outcomes.

### Statistical analysis

Data were analyzed using SPSS 26.0 software. All analyses were conducted according to the intention-to-treat principle. Continuous variables were expressed as mean ± standard deviation (x¯ ± S) and compared between groups using an independent samples t-test or Mann–Whitney U test. Categorical variables were presented as frequencies and percentages and analyzed using the chi-square test or Fisher's exact test, as appropriate. A *P*-value of <0.05 was considered statistically significant.

## Results

### Participants

**Figure 2:**
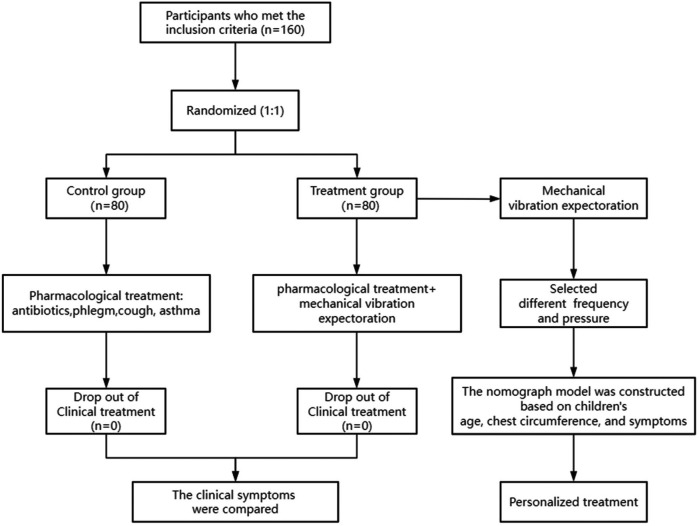
Study flowchart.

The study flowchart is shown in Figure 2. Our recruitment strategy involved collaboration with outpatient and inpatient departments to ensure a sufficient sample size. A total of 201 children diagnosed with pneumonia were recruited. Among them, 41 children were excluded (contraindications: *n* = 13, participating in other clinical trials: *n* = 28). Ultimately, 160 eligible patients were included and randomly assigned in a 1:1 ratio to the control group (*n* = 80) and the treatment group (*n* = 80). No participants withdrew from the study, and data collection was completed for all enrolled patients.

### Baseline characteristics

There were no statistically significant differences between the control and treatment groups in terms of gender, days of infection, respiratory rate, heart rate, age classification, chest circumference, moist rales, and disease severity, indicating that the two groups were comparable (Table 2).

**Table 2:** Baseline characteristics between the two groups of patients.

Variable	Category	All (*n* = 160)	Control group (*n* = 80)	Treatment group (*n* = 80)	*P*	Methods
Gender, *n* (%)	Female	67 (41.88)	37 (46.25)	30 (37.50)	0.262	Chi-square test
	Male	93 (58.13)	43 (53.75)	50 (62.50)		
Age classification, *n* (%)	1–3 years	16 (10.00)	4 (5.00)	12 (15.00)	0.055	Chi-square test
	4–8 years	114 (71.25)	63 (78.75)	51 (63.75)		
	9–14 years	30 (18.75)	13 (16.25)	17 (21.25)		
Moist rales, *n* (%)	Few	75 (46.88)	44 (55.00)	31 (38.75)	0.119	Chi-square test
	Moderate	55 (34.38)	23 (28.75)	32 (40.00)		
	Many	30 (18.75)	13 (16.25)	17 (21.25)		
Disease severity, *n* (%)	Mild	106 (66.25)	58 (72.50)	48 (60.00)	0.095	Chi-square test
	Severe	54 (33.75)	22 (27.50)	32 (40.00)		
Days of infection, median [IQR]	–	4.00 [4.00,4.00]	4.00 [4.00,4.00]	4.00 [4.00,4.00]	0.430	Mannwhitney-*U*
Respiratory rate, median [IQR]	–	23.00 [21.00,24.00]	23.00 [22.00,24.00]	22.00 [21.00,24.00]	0.101	Mannwhitney-*U*
Heart rate, mean (±SD)	–	85.50 ± 4.20	84.88 ± 4.00	86.13 ± 4.30	0.060	*t*-test
Chest circumference, mean (±SD)	–	61.65 ± 5.02	62.25 ± 4.33	61.05 ± 5.57	0.133	*t*-test

Units: Days of infection: day; Respiratory rate: beats/min; Heart rate: beats/min; Chest circumference: cm. Moist rales: Few: Sparse crackles heard in a limited lung field; Moderate: Diffuse crackles heard in multiple lung fields but not throughout the entire lung; Many: Extensive crackles present in nearly all lung fields.

### Clinical efficacy

After treatment, the clinical efficacy rate (patients achieving either a “markedly effective” or “effective” outcome) was 75% in the control group (pharmacological treatment alone) and 93.75% in the treatment group (pharmacological treatment combined with mechanical vibration-assisted sputum clearance therapy) (Table 3). The number of patients showing clinical improvement was significantly higher in the treatment group compared to the control group (*P* < 0.05), indicating that mechanical vibration-assisted sputum clearance therapy significantly enhanced the clinical efficacy compared to pharmacological treatment alone.

**Table 3:** Clinical efficacy between the two groups of patients.

Group	*N* (160)	Markedly effective, *n* (%)	Effective, *n* (%)	Ineffective, *n* (%)	Total effective rate, *n* (%)
Control group	80	28 (35.00)	32 (40.00)	20 (25.00)	60 (75.00)
Treatment group	80	54 (67.50)	21 (26.25)	5 (6.25)	75 (93.75)
*X* ^2^					10.667
P					0.001

### Comparison of clinical indicators between the two groups

Before treatment, there were no statistically significant differences between the two groups in terms of clinical symptoms, lung function, blood gas parameters, and inflammatory markers (Table 4). After treatment, significant improvements were observed in the treatment group compared to the control group. Specifically, the disappearance time of pulmonary rales was significantly shorter in the treatment group (5 days) than in the control group (8 days, *P* < 0.001). The time for fever resolution in the treatment group (2.38 ± 0.36 days) was also significantly shorter than that in the control group (3.32 ± 0.41 days, *P* < 0.001). Similarly, the cough resolution time in the treatment group (5 days) was shorter than in the control group (6 days, *P* < 0.001). The length of hospital stay was significantly reduced in the treatment group (6 days) compared to the control group (7 days, *P* < 0.001).

**Table 4:** Comparison of clinical indicators between the two groups of patients.

Variable	All (*n* = 160)	Control group (*n* = 80)	Treatment group (*n* = 80)	*P*	Methods
Disappearance time of cough, median [IQR]	6.00 [5.00,7.00]	6.00 [6.00,7.00]	5.00 [5.00,6.00]	<0.001	Mannwhitney-*U*
Disappearance time of pulmonary rales, median [IQR]	6.00 [5.00,8.00]	8.00 [6.00,8.00]	5.00 [5.00,6.00]	<0.001	Mannwhitney-*U*
Fever resolution time, mean (±SD)	2.85 ± 0.61	3.32 ± 0.41	2.38 ± 0.36	<0.001	*t*-test
Length of stay, median [IQR]	7.00 [6.00,8.00]	7.00 [7.00,8.00]	6.00 [5.00,7.00]	<0.001	Mannwhitney-*U*
FEV1 before, mean (±SD)	1.37 ± 0.30	1.35 ± 0.32	1.39 ± 0.27	0.301	*t*-test
FEV1 after, median[IQR]	2.10 [1.80,2.60]	1.80 [1.50,1.90]	2.60 [2.40,2.80]	<0.001	Mannwhitney-*U*
FEV1/FVC before, mean (±SD)	42.06 ± 7.01	41.01 ± 6.87	43.12 ± 6.99	0.058	*t*-test
FEV1/FVC after, mean (±SD)	65.34 ± 10.20	58.67 ± 7.66	72.02 ± 7.77	<0.001	*t*-test
pH before, mean (±SD)	7.21 ± 0.11	7.20 ± 0.08	7.23 ± 0.12	0.151	Welch's *t*-test
pH after, mean (±SD)	7.32 ± 0.14	7.29 ± 0.12	7.36 ± 0.15	0.001	*t*-test
PaCO_2_ before, median [IQR]	47.36 [44.82,49.76]	48.19 [44.93,50.01]	47.36 [44.75,49.01]	0.452	Mannwhitney-*U*
PaCO_2_ after, mean (±SD)	43.46 ± 3.13	44.62 ± 2.83	42.31 ± 3.00	<0.001	*t*-test
PaO_2_ before, median [IQR]	55.41 [53.47,58.27]	56.23 [53.58,58.78]	55.41 [53.47,57.99]	0.668	Mannwhitney-*U*
PaO_2_ after, mean (±SD)	79.70 ± 5.27	77.11 ± 4.49	82.30 ± 4.69	<0.001	*t*-test
CRP before, mean (±SD)	38.29 ± 6.09	37.56 ± 5.98	39.02 ± 6.11	0.131	*t*-test
CRP after, mean (±SD)	10.42 ± 2.82	13.02 ± 1.16	7.82 ± 1.02	<0.001	*t*-test
PCT before, mean (±SD)	0.63 ± 0.06	0.63 ± 0.05	0.64 ± 0.07	0.120	*t*-test
PCT after, mean (±SD)	0.42 ± 0.07	0.48 ± 0.04	0.36 ± 0.02	<0.001	Welch's *t*-test

Units: Length of stay, disappearance time of cough, fever clearance time, disappearance time of pulmonary rales: day.

Heart rate, respiratory rate: beats/min.

FEV1, FVC: L; FEV1/FVC: %.

PaO_2_, PaCO_2_: mmHg.

CRP: mg/L; PCT: μg/L.

In terms of lung function, the treatment group demonstrated a significant improvement in FEV1 (2.60 L) compared to the control group (1.80 L *P* < 0.001), and the FEV1/FVC ratio (72.02 ± 7.77%) was also higher than in the control group (58.67 ± 7.66%, *P* < 0.001). Regarding blood gas parameters, the treatment group exhibited a significantly higher PaO_2_ (82.30 ± 4.69 mmHg) than the control group (77.11 ± 4.49 mmHg, *P* < 0.001), while PaCO_2_ was lower in the treatment group (42.31 ± 3.00 mmHg) than in the control group (44.62 ± 2.83 mmHg, *P* < 0.001). The blood pH in the treatment group (7.36 ± 0.15) was also significantly higher than in the control group (7.29 ± 0.12, *P* = 0.001). Inflammatory marker levels were markedly reduced in the treatment group, with CRP levels significantly lower (7.82 ± 1.02 mg/L) than in the control group (13.02 ± 1.16 mg/L, *P* < 0.001), and PCT levels (0.36 ± 0.02 μg/L) were also lower than in the control group (0.48 ± 0.04 μg/L, *P* < 0.001). No adverse reactions were observed in either group.

### Variable selection and model construction

**Figure 3:**
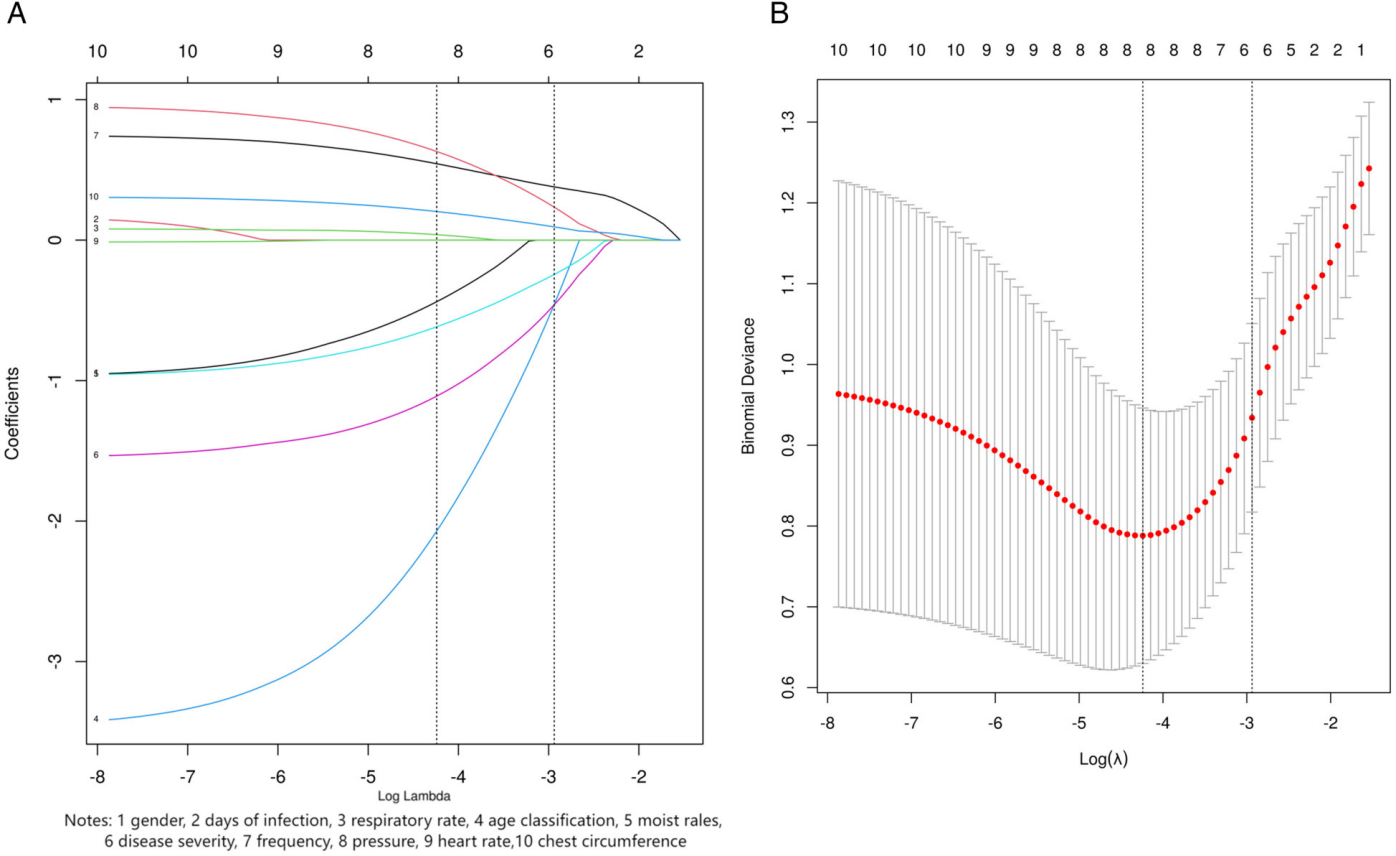
LASSO regression screening variables. **(A)** LASSO coefficient profile for ten candidate variables. **(B)** Lasso regression 10 fold cross validation curve. The dashed line in the right plot shows the optimal penalty value. In the left plot, its vertical intersects indicate the number of selected variables, with y-coordinates as their coefficients.

Based on the results of the Lasso regression analysis, six variables were selected as the most relevant predictors for clinical outcomes, including age classification, moist rales, disease severity, chest circumference, frequency, and pressure (Figure 3).

**Figure 4:**
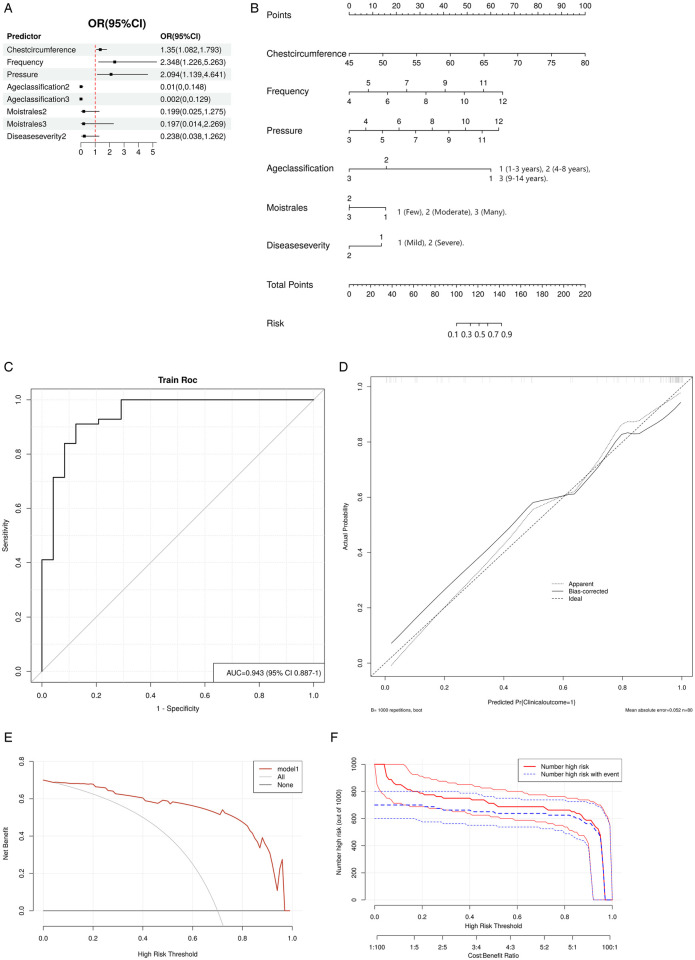
Logistic regression model. **(A)** Forest plot. **(B)** Nomogram. Point: Each variable is assigned a score based on its contribution to the model. Total point: The sum of all individual variable scores, which is used to predict the probability of improvement. **(C)** ROC curve. **(D)** Calibration curve. The dashed line represents the ideal result, the fine dashed line represents the observed result, and the solid line represents the bias-corrected result. **(E)** DCA curve. The black line indicates no improvement in clinical outcomes, and the red line represents the decision curve of the risk assessment model. **(F)** CIC curve. The *X*-axis of the CIC curve is the high risk threshold, the *Y*-axis is the number of people at risk in 1,000 people, the red line represents the number of people predicted by the model to occur the outcome event, and the blue line represents the actual number of people. The cost-benefit ratio at the bottom of the picture corresponds to the risk threshold.

Using these six variables, we constructed a forest plot (Figure 4A) and a nomogram model (Figure 4B) to predict the improvement of clinical outcomes in pediatric pneumonia treated with MVSC. The nomogram allowed clinicians to optimize the parameters of the MVSC device for personalized treatment. By inputting patient information, clinicians can obtain a predicted probability of clinical outcome improvement. For example, for a 5-year-old child with pneumonia, a chest circumference of 50 cm, mild moist rales, and mild disease severity, and using the device with a frequency of 7 Hz and pressure of 8 mmHg, the nomogram predicted total points of approximately 127.5, corresponding to a predicted probability of 70%. To increase the probability of improving the clinical outcome, the pressure and frequency of mechanical sputum discharge instruments can be appropriately increased.

The AUC for the nomogram was 0.943 (95% CI: 0.887–1), indicating excellent discrimination ability for predicting the clinical outcomes of pediatric pneumonia treated with MVSC (Figure 4C). The calibration plot demonstrated good consistency between the predicted probabilities and actual outcomes (Figure 4D). Additionally, the DCA (Figure 4E) revealed that the model had good clinical application value. CIC analysis (Figure 4F) revealed that when the threshold value was greater than 0.5, the model prediction and the actual occurrence were highly matched, and the clinical prediction efficiency was high.

In summary, this study not only confirmed the clinical effectiveness of MVSC but also developed a predictive model to optimize its therapeutic parameters. The primary function of the nomogram is to assist clinicians in refining the parameter settings of the mechanical vibration device, thereby providing personalized treatment plans tailored to individual patient characteristics. By integrating real-world patient data, the model enables evidence-based decision-making, allowing for precise adjustments to treatment parameters to maximize clinical benefits.

## Discussion

This study demonstrates the significant potential of MVSC therapy in improving clinical outcomes for pediatric pneumonia. By integrating individualized treatment parameters and developing an intelligent parameter selection model, we aimed to address limitations in traditional sputum clearance methods. The findings confirm the effectiveness of this approach, offering insights into its clinical application and broader implications for respiratory care in children.

Previous studies have shown that Maxing Ganshi Decoction could effectively improve the curative effect of the child's community-acquired pneumonia (*n* = 36). The clinical effective rate was 75% after 6 days of intervention and 88.88% after 10 days (18). Liu et al. (19) reported that N-acetylcysteine inhalation achieved a clinical efficacy rate of 94.83% in treating bronchopneumonia in children (*n* = 58), while ambroxol hydrochloride demonstrated an efficacy rate of 82.26% in a similar population (*n* = 62). Both treatments were administered via atomized inhalation for two weeks. Notably, in this study, Mechanical vibration-assisted therapy yielded a markedly higher clinical efficacy rate compared to standard pharmacological treatment (93.75% vs. 75%, *P* < 0.05). A clinical efficacy rate of 93.75% was achieved within a treatment duration of just five days. In addition, this was accomplished using a non-invasive auxiliary treatment that avoids potential harm to young patients, further highlighting its safety and feasibility. This improvement can be attributed to the proposed mechanisms of action, including enhanced mucus clearance (20), enhanced ciliary activity (21), and altered rheological properties of secretions (22). Specifically, chest wall oscillation (CWO) may play a crucial role in these processes by generating mechanical vibrations that enhance mucus mobilization and facilitate airway clearance, leading to faster symptom resolution. The application of CWO may produce rhythmic oscillations that help reduce mucus viscosity and disrupt secretion adhesions, potentially accelerating mucus movement toward the larger airways. Although mucus viscosity and sputum expectoration were not directly measured in this study, the mechanism of action of CWO suggests it could contribute to the observed reductions in pulmonary rales, possibly by decreasing airway secretion retention and thereby reducing the turbulence responsible for these abnormal sounds. Furthermore, CWO-induced improvements in expiratory flow and mucociliary clearance might facilitate faster resolution of cough symptoms, as more effective mucus clearance could help alleviate airway irritation and hypersensitivity. These mechanisms are particularly beneficial for young children, whose underdeveloped respiratory systems and narrow airways pose challenges for effective sputum clearance (23). Furthermore, the absence of adverse effects underscores its safety, making it a promising adjunctive treatment for pediatric pneumonia.

The therapy demonstrated significant benefits in improving blood gas parameters and reducing inflammatory markers. Post-treatment analysis revealed increased PaO_2_ and pH levels and decreased PaCO_2_ levels in the treatment group, indicating improved oxygenation and respiratory function. These findings suggest that enhanced mucus clearance restores ventilation-perfusion balance, a critical aspect of recovery in pneumonia (12). Similarly, the marked reductions in CRP and PCT levels highlight the therapy's role in mitigating systemic inflammation. By facilitating mucus clearance, mechanical vibration may reduce the bacterial load and inflammatory cytokine production in the respiratory tract, expediting the resolution of infection (24). This is particularly valuable in pediatric patients, who are more vulnerable to prolonged inflammation and its complications.

One of the key contributions of this study is the recognition of inter-individual variability among children and the corresponding need for personalized treatment approaches. Fixed vibration frequency and pressure settings fail to account for physiological differences such as age, chest circumference, and disease severity, which can significantly impact the effectiveness of sputum clearance. For example, younger children with smaller chest circumferences and narrower airways may require lower vibration frequency and pressure settings to achieve effective sputum clearance, whereas older children with larger chest sizes may tolerate and benefit from higher values. Inappropriate settings may compromise treatment efficacy and patient comfort, underscoring the critical need for individualized therapy. This study constructed the first nomogram model specifically designed to predict clinical outcome improvement in pediatric pneumonia treated with MVSC, achieving excellent predictive performance (AUC = 0.943). The model leveraged six key predictors—age classification, moist rales, disease severity, chest circumference, vibration frequency, and pressure—identified through LASSO regression analysis. By integrating these predictors, the nomogram offered clinicians a user-friendly tool to optimize treatment parameters tailored to patient-specific characteristics, bridging the gap between theoretical understanding and practical application. Previous studies have also employed nomogram models to predict outcomes in pediatric pneumonia, but these focused on different aspects of the disease. For example, Shen et al. (25) developed a nomogram to predict refractory Mycoplasma pneumonia in children, while Luo et al. (26) constructed a model for predicting outcomes in children with large-scale necrotizing pneumonia. Unlike these earlier works, our model is the first to specifically address mechanical vibration-assisted therapy, providing a framework for personalized parameter selection and highlighting its potential to enhance treatment precision and effectiveness. To facilitate clinical implementation, the nomogram could be embedded into electronic medical record (EMR) systems as a clinical decision support tool or developed into a mobile or web-based calculator. Such tools could assist clinicians in quickly determining optimal vibration settings based on patient-specific inputs (such as age and chest circumference), streamlining parameter adjustment, and improving bedside efficiency. Future efforts should focus on the development, validation, and usability testing of these digital implementations to enhance accessibility and clinical uptake.

Traditional sputum clearance techniques, such as postural drainage and chest percussion, have been widely used in pediatric pneumonia but face several limitations. These methods rely heavily on manual effort and technical expertise, leading to variability in effectiveness (27). Additionally, their reliance on patient cooperation can be challenging, particularly in young children. In contrast, mechanical vibration-assisted therapy offers a standardized, non-invasive, and reproducible approach that reduces dependency on manual techniques. The added benefit of real-time adjustability in vibration parameters further distinguishes this technology. By continuously optimizing frequency and pressure settings, clinicians can achieve higher efficacy while minimizing discomfort. The ability to integrate this technology into daily practice represents a significant improvement over traditional methods, particularly for hospitals with limited access to skilled personnel. The success of mechanical vibration-assisted therapy highlights its potential applications beyond pneumonia. Many pediatric respiratory diseases, such as bronchitis, cystic fibrosis, and asthma, involve mucus hypersecretion and impaired clearance. The principles demonstrated in this study could be adapted to these conditions, broadening the scope of this technology. Furthermore, the nomogram model could serve as a foundation for developing intelligent algorithms for other respiratory therapies, fostering a more personalized and data-driven approach to pediatric care.

This study has several limitations. First, the single-center design may limit the generalizability of the results. Future multi-center trials with larger and more diverse populations are needed to validate the findings and refine the nomogram model. Second, this study primarily focused on short-term clinical outcomes, such as symptom resolution and length of hospital stay, without evaluating long-term effects. The absence of follow-up data limits our understanding of the therapy's sustained impact. Future studies should be designed to evaluate long-term outcomes, including recurrence rates, chronic respiratory symptoms, and pulmonary function over time, to determine whether the observed benefits persist beyond the immediate treatment period. Lastly, although the nomogram demonstrated good performance in the internal dataset, internal validation using resampling or cross-validation methods was not performed for the predictive model, and external validation was also lacking. Future studies should incorporate external datasets to verify its generalizability.

## Conclusions

MVSC can improve the prognosis of children with pneumonia, and realize personalized treatment through an intelligent parameter selection model to solve the individual differences in children's physiology, providing a safe, effective, and non-invasive auxiliary means for traditional treatment.

## Data Availability

The original contributions presented in the study are included in the article/Supplementary Material, further inquiries can be directed to the corresponding author.
